# Supplementary material for: Establishment and maintenance of heritable chromatin structure during early Drosophila embryogenesis
Source: eLife. 2016 Nov 23;5:e20148. doi: 10.7554/eLife.20148 (PMC5156528; doi:10.7554/eLife.20148)
Supplement: Supplementary file 6. — DOI: http://dx.doi.org/10.7554/eLife.20148.042 [file elife-20148-supp6.pdf]

Normalization of ATAC-seq Data

Shelby Blythe

Autumn 2016

This markdown describes the general approach for normalization of coverage plots for 'open' ATAC seq data. It primarily serves to document how data was normalized in the accompanying manuscript. By making a few changes to the lines below (indicated), a user could perform identical normalization on their own using this code, if so desired.

What is needed at the outset is a set of mapped paired-end reads from one or more ATAC seq samples. In practice, these reads have been filtered by samtools for quality scores (q 30), and for PCR/Optical duplicates by Picard Mark Duplicates and samtools. These reads should be imported into R as a Genomic Ranges object, where each range represents the extent of the full sequenced fragment (GRanges start = 5' end of read 1, GRanges end = 5' end of read 2, assuming mate pairs are on opposite strands, which they should be). The start and end of each range is adjusted to reflect the original Tn5 interaction site, as described in Buenrostro 2013 (start -5, end +4).

The open 'peaks' used below, and .wig formatted versions of the standardized (z-scored) data are deposited in GEO (GSE83851)

Initial (CPM) Normalization

One thing that we want the data to tell us is for any region of the genome, how "accessible" is it? To estimate this, we will calculate the average number of reads coming from fragments derived from "open" chromatin within each 10 base-pair window across the entire genome. The starting data are a mixture of fragments from 'open' chromatin and 'nucleosome associated' chromatin. Although in this case we are only really interested in open chromatin, we observe that the ratio of open:nucleosome fragments varies depending on phase of the cell cycle. This means that if we were to perform simple counts per million (CPM) normalization of the data on only the open fragments, we would inflate the abundance of open fragments in samples with a smaller proportion of open fragments compared with nucleosome fragments. To account for this, we have used the total number of duplicate filtered, high quality reads as the denominator for the CPM normalization, rather than the total number of open fragments alone.

The following approach for counting average coverage per 10 bp window is an adaptation of scripts appearing in the following website:

> https://bioconductor.org/packages/devel/bioc/vignettes/GenomicRanges/inst/doc/GenomicRangesHOWTOs.pdf

First, we need a set of 10-bp windows from the dm6 build of the genome:

```
library( GenomicRanges)
library( GenomicAlignments)
library( BSgenome.Dmelanogaster.UCSC.dm6)

make.windows = function( binsize)
{
bins = tileGenome( seqinfo( Dmelanogaster), tilewidth = binsize,
                   cut.last.tile.in.chrom = TRUE)
return( bins)
```

```
}

genome.windows = make.windows( binsize = 10)
```

At this point, we should note that dm6 is a pain with its >1800 'chromosomes'. As a matter of practicality, we omit all non-canonical chromosomes. The following string will be used at several points to select only the 'good' chromosomes.

```
# note that in some cases, you may want 'chrY' as well, as this is helpful for
# deciding whether a single embryo is male or female. In practice, you can get
# away with simply measuring the X:Autosome ratio of total read coverage per
# chromosome length. chrY is only marginally useful, especially since the
# overall number of uniquely mapping reads will be significantly lower, and it
# will therefore be unlikely that you recover 1:1 read coverage between X and Y
# anyway.

good.uns = c( 'chr2L', 'chr2R', 'chr3L', 'chr3R', 'chr4', 'chrX')
```

Now that we have defined those variables, we load a dataset. The following line should be changed to reflect a file on your own computer.

```
load( '/Volumes/seq_data/ATAC/Complete_Timecourse_Analysis_Sets/all_bamfiles/GRanges/WT_15042105_NC13_1[illegible]

# this loads a dataset used in the accompanying manuscript, corresponding to a
# single embryo. The next line simply assigns the stored variable name of this
# dataset to something more descriptive for the purposes of this demonstration.

all.data = x
```

We will want to use the total number of reads in *all.data* as the denominator for CPM normalization.

```
total.reads = length( all.data)

total.reads
```

```
## [1] 6922040
```

The distribution of read-lengths can be plotted in order to get an idea of the relative fraction of small, open reads

```
hist( width( all.data), breaks = 500, main = "Read-length distribution for
     'all.data'", xlab = 'length (bp)', xaxt = 'n', xlim = c( 0,1000))
axis(1, at = seq( 0, 1000, by = 100))

abline( v = 98, col = 'red', lwd = 2, lty = 3)
```

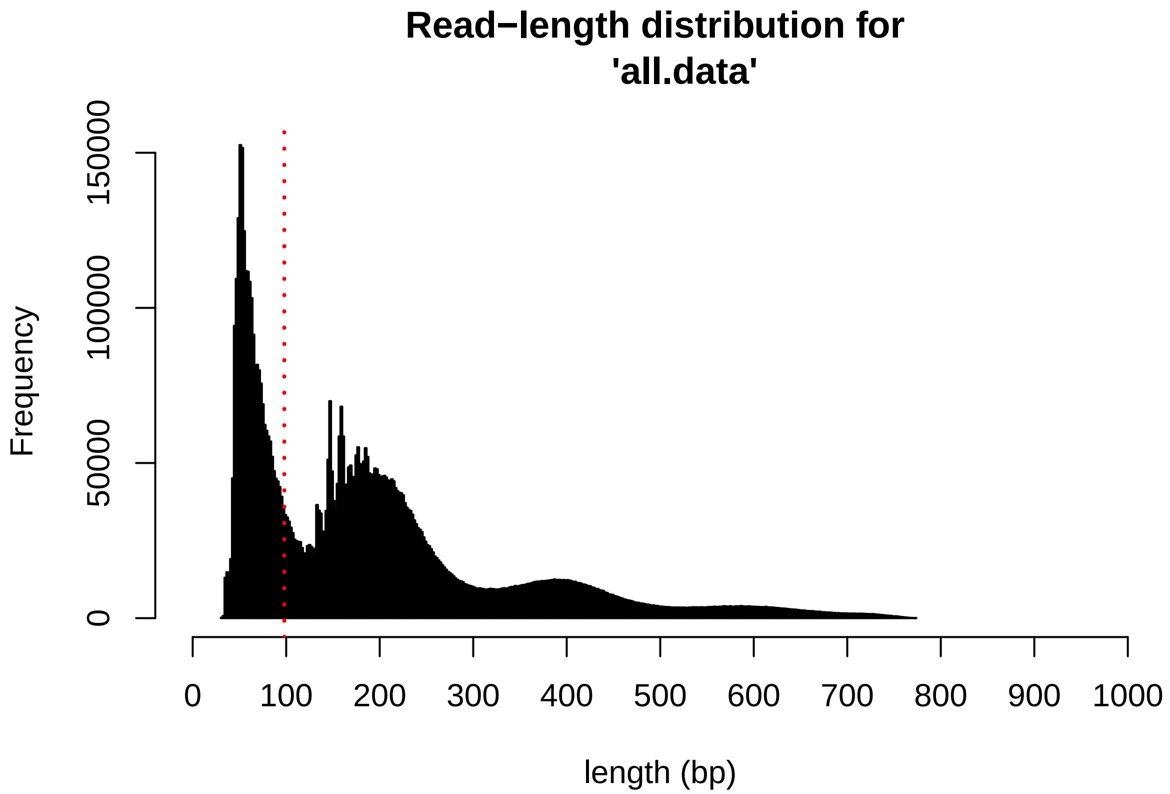

You can see that that a significant proportion of the fragments are present within the small, open read (< or = 98 bp) range of the distribution. Although it appears that there may be more small reads to the right of the red line, elsewhere I have fit these distributions to a single exponential and three gaussians and found that 98 bp is the greatest size that I can use, in this dataset, to declare a region to be open, and have less than a 5% likelihood of a read actually belonging to the +1 nucleosome distribution to the right. With different datasets, this cutoff may be different. I believe Buenrostro 2013 used a 100 bp cutoff which would probably be fine as well.

We now define the set of *open.reads* that correspond to the < or = 98 bp fragments.

```
open.reads = all.data[width( all.data) <= 98]
```

Next, use the following lines to calculate the average number of reads within a 10 bp window.

```
basecount = function( windows, data, chrlist)
{
    # this is an instance of the general basecount function that takes as input
    # a set of bins (in GRanges format), a GRanges object containing count data,
    # and a list of chromosomes of interest. It returns a GRanges object
    # identical to the input bins, with a score column indicating the average
    # number of instances where a feature in the count data was present within
    # the bin. It does not normalize to anything.
    require( GenomicRanges)
    require( GenomicAlignments)

    binnedAverage = function( bins,numvar)
    {
        stopifnot( is( bins, "GRanges"))
        stopifnot( is( numvar, "RleList"))
```

```
        stopifnot( identical( seqlevels( bins), names( numvar)))
        bins_per_chrom = split( ranges( bins), seqnames( bins))
        means_list = lapply( names( numvar),
            function( seqname){
                views = Views( numvar[[seqname]],
                bins_per_chrom[[seqname]])
            viewMeans( views)
            })
        new_mcol = unsplit( means_list, as.factor( seqnames( bins)))
        mcols( bins)[['basecount']] = new_mcol
        bins
    }

    good.uns = chrlist
    windows = windows[seqnames( windows) %in% good.uns]
    seqlevels( windows) = seqlevelsInUse( windows)
    cov = coverage( data)
    cov = cov[match( seqlevels( windows),names( cov))]

    bcount = binnedAverage( windows, cov)
    bcount = mcols( bcount)[,1]
    score( windows) = bcount
    return( windows)

}

# now we execute the function to make the initial coverage count:

open.basecount = basecount( windows = genome.windows, data = open.reads,
                            chrlist = good.uns)

# note that the metadata column 'score' is the raw average value, and we still
# need to normalize it to the total number of reads in the entire dataset.

score( open.basecount) = score( open.basecount) / total.reads * 1e+6
```

Standardization of Data

In the associated manuscript, what I've done is performed ATAC seq on multiple related biological samples, and part of the analysis requires comparison between these replicates.

The following line loads a GRangesList object that the user will have to generate independently, which consists of the output of the above section (CPM normalized average coverage in 10 bp windows), applied to each of the timepoints measured for wild-type embryos in the associated manuscript. Each timepoint consisted of at least three independent biological replicates, and for the purposes of analysis, all replicates for each timepoint were pooled together prior to CPM normalization.

```
load("/Volumes/seq_data/ATAC/Complete_Timecourse_Analysis_Sets/open_basecounts_nodups/wt.open.v2.baseco[illegible]

# this opens a saved object named wt.open
```

As part of the analysis in the associated manuscript, I have called peaks on these data and these represent

regions of interest for further analysis. We'd like to compare reads in each of these regions.

The following line loads these up in GRanges format. The user will have to point the next line to the appropriate file on their own computer.

```
load( '/Volumes/seq_data/ATAC/Complete_Timecourse_Analysis_Sets/New_Peaks_Lists/ATAC.open.peaks')

# this opens a saved object named ATAC.open.peaks

ATAC.open.peaks = ATAC.open.peaks[!seqnames( ATAC.open.peaks) %in% 'chrY']
# this version I loaded contains some peaks in chrY that were not considered in
# the official peaks list from the associated manuscript.
```

Like with any genomic dataset, the relative proportion of 'high coverage' 10 bp windows is a small fraction of the total number of 10 bp windows. The majority of these high coverage windows are captured in the peaks list. In order to compare how the counts within peaks compare across timepoints, I have chosen to further standardize the coverage scores.

Standardization transforms the coverage values from counts per million to (effectively) the number of standard deviations above or below the population mean. To do this, I calculate the average number of CPM in a set of noisy or background regions, and then scale the entire dataset so that these background regions have a mean value of zero and a standard deviation of one.

The following section illustrates how this is done in practice.

```
# the following section designates a set of official 'noise' peaks from which
# to determine background counts.

N = 2.5e+4              # the number of background regions to generate

bins = genome.windows[seqnames( genome.windows) %in% good.uns]
# create a container for the standardized data

notopen = gaps(
    reduce(
        trim(
            resize( ATAC.open.peaks, fix = 'center', width = 20000))))

notopen = notopen[strand( notopen) %in% '*']

# the above lines define which genome windows do not overlap with the peaks,
# I have enlarged the peaks to a uniform 20000 bp each and reduced overlaps to
# single intervals prior to identifying gaps in peak coverage. The second line
# is there because gaps() returns results on all three strand types, but we only
# need the * strand.


bgoverI = subjectHits( findOverlaps( notopen, bins))
# this finds the indices of 10 bp windows overlapping the set of noise ranges.

set.seed(138) # sets the seed for random number selection.

bgseeds = sort( bgoverI[sample( seq( 1,length( bgoverI),1),N)])
# this samples N of the noise regions for use downstream
```

```r
bgpeaks = trim(
    resize(
        bins[bgseeds],
        width = 2^( rnorm( N, mean = mean( log2( width( ATAC.open.peaks))),
                           sd = sd( log2( width(ATAC.open.peaks))))),
        fix = 'center'))

# this models a set of noise peaks whose centers are the regions we sampled
# above, and whose widths are randomly distributed over the mean and sd of the
# log2 widths of the true peaks list. The log2 widths of the peaks approximates
# a normal distribution.

bgpeaks = reduce(
    bgpeaks[!as.logical( countOverlaps( bgpeaks, ATAC.open.peaks))])

# sometimes the peaks we've randomly picked overlap with one another. the way I
# handle this is to reduce them to single intervals rather than count them twice.

# Now, we need to measure the mean and standard deviation of reads within this
# set of background regions for each timepoint.


bgmeans = NULL # container for measured means
bgsds = NULL # container for measured standard deviations
openmeans = NULL # container for the measured open means
opensds = NULL # container for the measured open sds
zscores = list() # container for the calculated z-scores
bgzmeans = NULL # container for the standardized means
bgzsds = NULL # container for the standardized standard deviations
openzmeans = NULL # container for the standardized open means
openzsds = NULL # container for the standardized open sds

for(i in 1 : length( wt.open))
{
    #cat( paste0( names( wt.open)[i], ' ')) # just a progress indicator

    bgoverscores = score(
        wt.open[[i]])[subjectHits( findOverlaps( bgpeaks, bins))]
    # this gets all of the scores overlapping with one of the noise peaks

    openoverscores = score(
        wt.open[[i]])[subjectHits( findOverlaps( ATAC.open.peaks, bins))]

    bgmean = mean( bgoverscores) # calculates the mean of these scores
    bgsd = sd( bgoverscores) # calculates the standard deviation of these scores
    bgmeans[i] = bgmean # assigns the mean to our initialized object
    bgsds[i] = bgsd # assigns the sd to our initialized object
    openmeans[i] = mean( openoverscores) # calculates the mean of the open scores
    opensds[i] = sd( openoverscores) # calculates the mean of the open sds

    znorm = score( wt.open[[i]]) # populates a new variable with the scores
    znorm = ( znorm - bgmean) / bgsd # calculates the zscore
    zscores[[i]] = znorm # populates our initialized object with the vector
```

```
    # of z-scores for i-th timepoint

    bgz = znorm[subjectHits( findOverlaps( bgpeaks, bins))]
    # gets the new calculated z-scores overlapping the noise peaks

    openz = znorm[subjectHits( findOverlaps( ATAC.open.peaks, bins))]
    # gets the new calculated z-scores overlapping the open peaks

    bgzmeans[i] = mean( bgz) # populates our initialized object with the z mean
    bgzsds[i] = sd( bgz) # populates our initialized object with the z sd
    openzmeans[i] = mean( openz) # populates our initialized object with z mean
    # for open regions
    openzsds[i] = sd( openz) # populates our initialized object with the z sd
    # for open regions

}

# what does this do to the data?

initial.mean = unlist( lapply( wt.open, function( x) mean( score( x))))
zscore.mean = unlist( lapply( zscores, function( x) mean( x)))

stripchart(
    list( initial.mean, zscore.mean, bgmeans, bgzmeans, openmeans, openzmeans),
    vertical = TRUE, pch = 19, col = 'blue', method = 'jitter', las = 1,
    xlab = '', xaxt = 'n', ylab = 'mean score per timepoint')

mtext(side = 1, at = c(1:6),
      c( 'CPM.all', 'Z.all','BG.CPM', 'BG.Z', 'OPEN.CPM','OPEN.Z'))

boxplot(
    list( initial.mean, zscore.mean, bgmeans, bgzmeans, openmeans, openzmeans),
    add = TRUE, xaxt = 'n', yaxt = 'n')
```

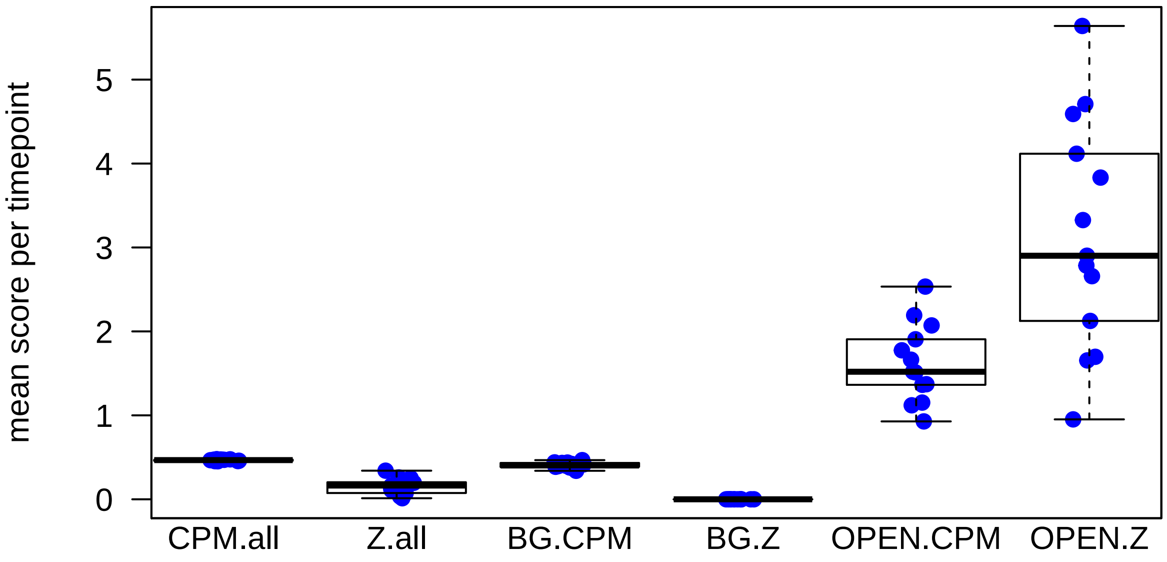

The new z-scores can be appended to the *wt.open* object as a score and used for further analysis. Note, the boxplot above is meant to highlight how the average values of the scores change with this normalization. It is

important to keep in mind that the effective units for the CPM values are in counts per million, whereas the z-score units are in standard deviations above the mean.

Session Info

```r
sessionInfo()
```

```
## R version 3.3.1 (2016-06-21)
## Platform: x86_64-apple-darwin13.4.0 (64-bit)
## Running under: OS X 10.11.3 (El Capitan)
##
## locale:
## [1] en_US.UTF-8/en_US.UTF-8/en_US.UTF-8/C/en_US.UTF-8/en_US.UTF-8
##
## attached base packages:
## [1] parallel  stats4    stats     graphics  grDevices utils     datasets
## [8] methods   base
##
## other attached packages:
##  [1] BSgenome.Dmelanogaster.UCSC.dm6_1.4.1
##  [2] BSgenome_1.42.0
##  [3] rtracklayer_1.34.0
##  [4] GenomicAlignments_1.10.0
##  [5] Rsamtools_1.26.0
##  [6] Biostrings_2.42.0
##  [7] XVector_0.14.0
##  [8] SummarizedExperiment_1.4.0
##  [9] Biobase_2.34.0
## [10] GenomicRanges_1.26.0
## [11] GenomeInfoDb_1.10.0
## [12] IRanges_2.8.0
## [13] S4Vectors_0.12.0
## [14] BiocGenerics_0.20.0
##
## loaded via a namespace (and not attached):
##  [1] Rcpp_0.12.7        knitr_1.14         magrittr_1.5
##  [4] zlibbioc_1.20.0    BiocParallel_1.8.0 lattice_0.20-33
##  [7] stringr_1.1.0      tools_3.3.1        grid_3.3.1
## [10] htmltools_0.3.5    yaml_2.1.13        assertthat_0.1
## [13] digest_0.6.10      tibble_1.2         Matrix_1.2-6
## [16] formatR_1.4        bitops_1.0-6       RCurl_1.95-4.8
## [19] evaluate_0.10      rmarkdown_1.1      stringi_1.1.2
## [22] XML_3.98-1.4
```
